# Why the Register Must Fail from Incompleteness

**Published:** 1908-12-12

**Authors:** 


### Nursing Administration.

### WHY THE REGISTER MUST FAIL FROM INCOMPLETENESS.

Among all the difficulties which would await the Council of Registration, the task of keeping the register must be taken into serious account. It may be considered that the success and public value to which a Register can attain in any profession are measured by the completeness with which it can gather together the members under its protection. The value of the Medical Register, for instance, consists in the fact that it embraces the entire medical profession, and that no man can hope to build up a reputable private practice unless his name is to be found in it. As soon as an influential section of people in any profession elect to remain outside a register, half its value is gone. For the public is faced with the dilemma that certain persons outside the register are more highly qualified than a certain section within. And thus confusion is introduced in place of order. Is there any reasonable prospect that a register of trained nurses could be made, we will not say complete, but even tolerably representative?

It has been estimated that there are about 20,000 nurses whose claim to have received training might admit them to the register. We are not concerned with the exactness of these figures. The number might easily be greater, since the output of probationers turned out annually by the larger training schools is now (if Poor-law infirmaries be included) probably not less than 1,500. What proportion of these 20,000 nurses would pay a fee, would make application even if no fee were demanded, to appear in the register? In the first place, few who have given up nursing from marriage or other causes would do so, yet many of these frequently take occasional cases when they come in their way, and it is among these stragglers from the main body that abuses are most likely to creep in. Hundreds of nurses, with all the publicity which would be accorded to it, would never get to hear about the register. Hundreds more would never get to understand what they were expected to do. Hundreds in permanent posts would declare it was not of any use to them. No one who has had any experience in keeping nurses together can believe that even a bare majority of trained nurses would enter their names on the list. But this is but the beginning of difficulties. All nurses, even those trained in the best hospitals in the kingdom, are accustomed to regard their subsequent posts as additional training, and the entries after a nurse's name which she would deem essential to the full understanding of her worth and experience might well stretch to the number of ten or twelve. The point then arises: will the State Register ignore all the nurse's doings subsequent to her being admitted to the register, or will the register be compelled to undertake the duty of verifying such additional details regarding her work as the nurse may desire to have recorded to her credit? If the particulars entered to the name of a nurse be limited to the bare years of training which have qualified her for registration, we have no hesitation in saying that a large proportion of nurses will consider themselves to be deprived thereby of their best credentials. For a nurse trained in a county infirmary may subsequently hold a responsible position in some other institution which would entitle her to specialist work of the most remunerative description. Would she not have just cause for complaint if the training she shared in common with hundreds of other candidates were entered against her name to the exclusion of those particular qualifications on which her income depended? If, on the other hand, the Registrar is to be charged with the duty of entering "supplementary information" with regard to such nurses as may desire to pay an extra fee for this purpose, applications must perforce be made to the matron or secretary of every hospital with which the candidate has been connected. Among the older nurses the information required before any clear impression could be offered to the public of their career (and a nurse's qualifications are inextricably bound up in her career) would be bewildering in extent, and, what is most disheartening, the information once obtained and the register brought out, after hundreds of thousands of verification slips had been sorted and printed at enormous expense, much of it would be out of date before it could be issued. The constant change of nurses from one place to another makes the task of keeping them in touch with a central bureau stupendous, and each change in situation involves correction, fresh expense, fresh liability to error. We fear that the register, after every possible precaution had been taken, could not fail in regard to accuracy and completeness to be a matter of disappointment to all.

In order to avoid the peril of keeping on the register the names of nurses who have given up nursing, married, or died, the very necessary provision has been made in the Nurses' Registration Bill, that every registered nurse must pay a fee of 2s. 6d. every year for the privilege of renewing her name. Should she fail to do this her name may be removed from the register, and will not then be restored except on payment of 5s., after a reasonable excuse has been proffered and accepted by the authorities. While admitting the necessity for this regulation, unless the register is to be cumbered with misleading entries, we would call attention to the irritating character of this annual demand to a body of women so scattered and arduously engaged as nurses. The difficulty of obtaining verification slips even from professional people of settled habits and address is notorious. The difficulties in the case of nurses are multiplied ten-fold. While a mere fraction of the trained nurses of the country would, we fear, go through the necessary formalities to qualify for admission to the register, it can be very distinctly foreseen that only a mere fraction of the registered would take the necessary steps year by year to keep their names on the books, and among this fraction would certainly be numbered registered nurses with little else to recommend them. The failure of the register as a work of reference is a foregone conclusion.

